# Platelet-Activating Factor Induces TLR4 Expression in Intestinal Epithelial Cells: Implication for the Pathogenesis of Necrotizing Enterocolitis

**DOI:** 10.1371/journal.pone.0015044

**Published:** 2010-10-15

**Authors:** Antoine Soliman, Kathrin S. Michelsen, Hisae Karahashi, Jing Lu, Fan Jing Meng, Xiaowu Qu, Timothy R. Crother, Shervin Rabizadeh, Shuang Chen, Michael S. Caplan, Moshe Arditi, Tamas Jilling

**Affiliations:** 1 Department of Pediatrics, University of California Los Angeles School of Medicine, Los Angeles, California, United States of America; 2 Department of Pediatrics, Northwestern University Feinberg School of Medicine, Chicago, Illinois, United States of America; 3 Evanston Northwestern Healthcare Research Institute, Evanston, Illinois, United States of America; New York University, United States of America

## Abstract

Necrotizing enterocolitis (NEC) is a leading cause of morbidity and mortality in neonatal intensive care units, however its pathogenesis is not completely understood. We have previously shown that platelet activating factor (PAF), bacteria and TLR4 are all important factors in the development of NEC. Given that Toll-like receptors (TLRs) are expressed at low levels in enterocytes of the mature gastrointestinal tract, but were shown to be aberrantly over-expressed in enterocytes in experimental NEC, we examined the regulation of TLR4 expression and signaling by PAF in intestinal epithelial cells using human and mouse in vitro cell lines, and the ex vivo rat intestinal loop model. In intestinal epithelial cell (IEC) lines, PAF stimulation yielded upregulation of both TLR4 mRNA and protein expression and led to increased IL-8 secretion following stimulation with LPS (in an otherwise LPS minimally responsive cell line). PAF stimulation resulted in increased human TLR4 promoter activation in a dose dependent manner. Western blotting and immunohistochemical analysis showed PAF induced STAT3 phosphorylation and nuclear translocation in IEC, and PAF-induced TLR4 expression was inhibited by STAT3 and NFκB Inhibitors. Our findings provide evidence for a mechanism by which PAF augments inflammation in the intestinal epithelium through abnormal TLR4 upregulation, thereby contributing to the intestinal injury of NEC.

## Introduction

Necrotizing enterocolitis (NEC) is a devastating gastrointestinal disease primarily affecting premature infants, and despite recent advances in neonatal intensive care, it remains a leading cause of morbidity and mortality in this high-risk population.

The etiology of NEC remains elusive, but inflammation is thought to be central to its pathogenesis, leading to intestinal injury and associated morbidities [1], [2], [3], [4]. Previously, we and others have shown that platelet activating factor plays a key role in NEC in humans and in animals [5], [6], [7], [8], [9], [10], [11], [12]. Platelet activating factor (PAF) is a potent endogenous, extracellular and intracellular phospholipid mediator, produced by a two-step enzymatic process where phosphatidyl choline is the de novo precursor and phospholipase A_2_ (PLA_2_-II) is the rate-limiting enzyme. The secreted form of PLA_2_-II, responsible for the extracellular production of PAF, is expressed by many cell types including intestinal paneth cells and its release is triggered by hypoxia, endotoxemia, and trauma among other stimuli. Activation of its G protein-coupled receptor, PAF-receptor (PAFR), leads to activation of several signal transduction pathways including the signal transducers and activators of transcription (STATs) and NFκB [13], culminating in physiological or pathological changes including vasoconstriction and/or vasodilatation, leukocyte stimulation and migration, synthesis and activation of cell adhesion molecules, increased capillary permeability, production of reactive oxygen and nitrogen species, and alterations in intestinal mucosal permeability [14], [15].

In addition to PAF, the presence of enteric bacteria appears to be a prerequisite for development of NEC [16]. Indeed, antibiotic use has been shown to have a protective role in NEC in the rodent model [17]. Enteric bacteria trigger Toll-like receptors (TLRs), a family of transmembrane molecules that recognize specific repetitive patterns associated with bacterial products [18], and both bacteria and TLRs have been shown to be important for experimental NEC pathogenesis [16], [19]. However, we [20], [21] and others [22] have shown that the healthy, adult human intestinal epithelium expresses low levels of TLR2 and TLR4, which may serve to limit intestinal inflammation and injury in the face of normal intraluminal bacterial exposure. On the other hand, this low level expression is subject to regulation by some inflammatory mediators (36).

We hypothesized that PAF, which is released due to hypoxia, infection, or local injury, induces an upregulation of TLR4 at the intestinal epithelium that predisposes to excessive bacterial activation of the intestinal inflammatory response, leading to intestinal necrosis and NEC. We have found that in various human and rodent tissue culture models of intestinal epithelium, stimulation with PAF results in an upregulation of TLR4 expression, correlating with an enhanced TLR4 ligand-induced inflammatory gene expression. Furthermore, in cells over-expressing PAFR, treatment with PAF resulted in a dose-dependent activation of a TLR4 promoter reporter construct, and in an *in vivo* model, luminal perfusion of an ileal loop with PAF resulted in enhanced intestinal TLR4 gene expression. PAF caused a translocation of STAT3 from the cytosol to the nuclei of enterocytes and the PAF-induced induction of TLR4 expression was inhibited by pharmacological inhibition of the STAT3 and NFkB signaling pathways.

## Materials and Methods

### Ethics Statement

All experiments were performed according to the guidelines and approved protocol (IACUC approval ID #EH05-026) of the NorthShore University HealthSystem Institutional Animal Care and Use Committee and were housed under specific pathogen free conditions.

### Reagents and Inhibitors

Carbamyl PAF C-16 (cPAF) (Alexis Biochemicals, San Diego, CA), dissolved in ethanol, was used in stimulation of cells. Highly purified, phenol-water-extracted *Escherichia coli* K235 LPS (<0.008% protein) was prepared according to the method of McIntire et al. [23] and obtained from S. N. Vogel (Uniformed Services University of the Health Sciences, Bethesda, MD) [24], [25]. The purity of this LPS has been demonstrated previously [26], [27]. The following inhibitors were used to pretreat IEC-6 cells for 30 minutes prior to and throughout PAF stimulation (all from Calbiochem/EMD Biosciences, San Diego, CA): BAY 11–7082 to inhibit nuclear translocation of NFκB, Proteasome Inhibitor II (PS I–II), Tyrophostin AG490 protein kinase inhibitor that selectively inhibits activation of STAT3.

### Cell culture

Human embryonic kidney cells (HEK293), COS-7 cells, human intestinal epithelial Caco-2 and rat small intestine epithelial cell line IEC-6 were obtained from the American Type Culture Collection (ATCC, Manassas, VA), and were grown as recommended by ATCC. The HT29-CL19A, differentiated colonocyte cell line was a generous gift from C.L. Laboisse (Cleveland, OH) [28] and were maintained in DMEM with 10% FBS, 2% Pen/Strep. For experiments that required transfection, cells were set up at 30% confluence and were transfected using Lipofectamine 2000 according to the manufacturer's instructions.

### Intraluminal perfusion of PAF in isolated ileal loop

Following 24 hrs of fasting (water only), juvenile rats (approx 150 g body weight) were anesthetized with Nembutal. Following cannulation of the left jugular vein, the right carotid artery, and the trachea, a midline abdominal incision was made. The cecum was brought to the surface and PE-50 tubing was secured into ileum just proximal to the cecum. Following inflation of the intestine with 2 ml saline (vehicle) or 2 ml PAF at various doses, the intestine was lowered in the abdominal cavity, and the abdominal wall was closed around the PE tubing. While maintaining anesthesia, the intestine was perfused for 4–6 hrs with saline, or saline + PAF etc. at a rate of 1 ml/h. Hematocrit was measured every 30 minutes. At the termination of the experiment animals were euthanized with pentobarbital, intestines are collected and split in half lengthwise, half for frozen sectioning and the other half for RNA isolation.

### Construct propagation and purification

The human TLR4 promoter-luciferase cDNA was a kind gift from M. Rehli (Regensburg, Germany), preparation previously described [29]. pCMV-β-galactosidase were used as previously described [20]. Human platelet activating factor receptor (PAFR) adenoviral vectors were constructed using the AdEasy system (Stratagene, La Jolla, CA) as recommended by the manufacturer. Viruses were purified through serial CsCl density gradient centrifugations and subsequent dialysis. Viral titers were quantified by infecting HEK293 cells with serial dilutions of the virus. All viral preparations were screened for Endotoxin contaminations (Endosafe, Charles River Laboratories, MA). Additionally, cDNA corresponding to tagged wild type human PAFR was subcloned downstream from a human immediate-early CMV promoter-enhancer element into the pCDNA 3.1/GS vector (Invitrogen, Carlsbad, CA).

### Transient gene expression and reporter gene assays

HEK293 cells were co-transfected with CMV-β-galactosidase (0.075 µg), PAFR constructs (0.2 µg), and human TLR4 promoter-luciferase (0.05 µg) using FuGENE 6 transfection reagent (Roche, Basel, Switzerland) as per the manufacturer's instructions in 24 well plates. After overnight transfection, cells were stimulated for 5 h with cPAF (0–150 nM, as indicated), and luciferase activity was measured with a luciferase kit (Promega, Madison, WI) as described previously [30]. Transfection efficiency was normalized by assaying for β-galactosidase activity using a colorimetric method (Stratagene, La Jolla, CA) as previously described [30].

### Quantitative real time PCR

Transcript levels were determined using QRT-PCR normalized to GAPDH. Primers and probes used: rat GAPDH-VIC, human GAPDH-VIC (both primers limited) with rat and human TLR4-6-FAM primer and probe sets (Applied Biosystems, Foster City, CA). IEC-6 cell RNA was isolated using RNA STAT-60 (Tel-Test Inc, Friendswood, TX) and from Caco-2 cells using RNeasy columns (Qiagen, Valencia, CA). RNA was quantified and integrity was verified by agarose gel electrophoresis. cDNA was prepared by reverse transcription from 1 µg of IEC-6 of RNA and 25 ng of Caco-2 RNA. For quantification of TLR4 in IEC-6, each 25 µl QRT-PCR reaction contained 1 µl of cDNA, 900 nM each primer, 250 nM probe universal PCR master mix (Applied Biosystems, Foster City, CA). These QRT-PCR reactions were preformed in a Smartcycler (Cepheid, Sunnyvale, CA). For Caco-2 cells, each 10 µl QRT-PCR reaction contained 0.5 µl cDNA, 900 nM primer, 250 nM probe (both GAPDH and TLR4 probes in a duplex reaction) and Jump Start TAQ Ready Mix PCR master mix (Sigma, St, Louis MO) according to manufacturer's suggested methods. These QRT-PCR were performed in a Rotor Gene machine (Corbett Life Science, Sydney, Australia). Each primer and probe set was initially analyzed and found to have a linear relationship between 2^−CT^, and the dilution factor for all reactions showed CT values <38.

### Nuclear and cytoplasmic fraction preparation and western blotting

IEC-6 cells were grown to confluence in 10 cm Petri dishes and were treated with or without cPAF as indicated. Cells were harvested and nuclear and cytoplasmic fractions were prepared using a Nuclear Extract kit from Active Motif (Carlsbad, CA). Samples were subjected to SDS-PAGE and transferred to PVDF membrane, blocked with 5% non-fat milk in TBST-0.1% Tween 20 and incubated with mouse monoclonal antibodies against STAT3 (Invitrogen/Zymed, Carlsbad, CA) followed by horseradish peroxidase-conjugated anti-mouse IgG (Santa Cruz Biotechnology, Santa Cruz, CA), or were incubated with a rabbit polyclonal antibody to STAT3 pY705, (Biomol, Plymouth Meeting, PA), followed by incubation with horseradish peroxidase-conjugated anti-rabbit IgG (Santa Cruz Biotechnology, CA). Blots were visualized using enhanced chemiluminescence detection solution (Amersham Pharmacia Biotech, Piscataway, NJ) and scanned with a Phosphor Imager (Molecular Dynamics; Piscataway, NJ).

### Immunocytochemistry

For immunocytochemistry analysis, IEC-6, COS-7 or HT29-CL19A cells were seeded onto glass coverslips. COS-7 cells were exposed to AD-PAFR (10^9^ viral particles/10^6^ cells) for 24 hrs. All cells were treated with cPAF or vehicle as indicated. COS-7 cells were fixed in 4% paraformaldehyde, all other cells were fixed in 100% methanol at −20°C, and all cells permeabilized with 0.1% Triton X-100 in PBS. Following washing and blocking, cells were probed with mouse monoclonal anti-STAT3 (diluted 1∶50 in 10% goat serum) (Invitrogen/Molecular Probes, Carlsbad, CA) overnight at 4°C in a humidified chamber followed by incubation with Alexa Fluor 546 labeled goat anti-mouse secondary antibody for 1 h at 37°C in a humidified chamber. Coverslips were mounted onto glass slides in anti-fade mounting medium (Molecular Probes) and observed by fluorescence microscopy.

### Image analysis and display

Specimens were viewed using a Leica microscope equipped with epifluorescence illumination and appropriate fluorescence filters for DAPI, Alexa Fluor 488/GFP and Alexa Fluor 546. Fluorescence images in each wavelength were collected using a Coolsnap CCD camera and were relayed to the IPLab Spectrum image analysis software. Images from the control and experimental groups were subjected to identical grayscale normalization and contrast enhancement and were then subjected to pseudocolor overlay to produce the color images.

### ELISA

For human IL-8 detection, 50,000 Caco-2 cells were plated per well in 96-well plates. Cells were treated with cPAF (5 µM) for 24 h, then washed with PBS and stimulated with LPS (10 ng/ml) overnight. The supernatants were harvested for measurement of IL-8 using IL-8 ELISA Kit (R&D Systems, Minneapolis, MN) as per the manufacturer's instructions.

### Statistical Analysis

Data are reported as mean values ±S.D of three or more independent experiments. The statistical significance of differences between mean values was determined by Student's *t* test, unless otherwise indicated. Student's *t* tests, standard deviation, and standard errors were performed using the statistics package within Microsoft Excel. A *p* value of less than 0.05 was considered significant.

## Results

### Platelet-activating factor induces TLR4 expression in the rat intestinal mucosa

**Figure 1:**
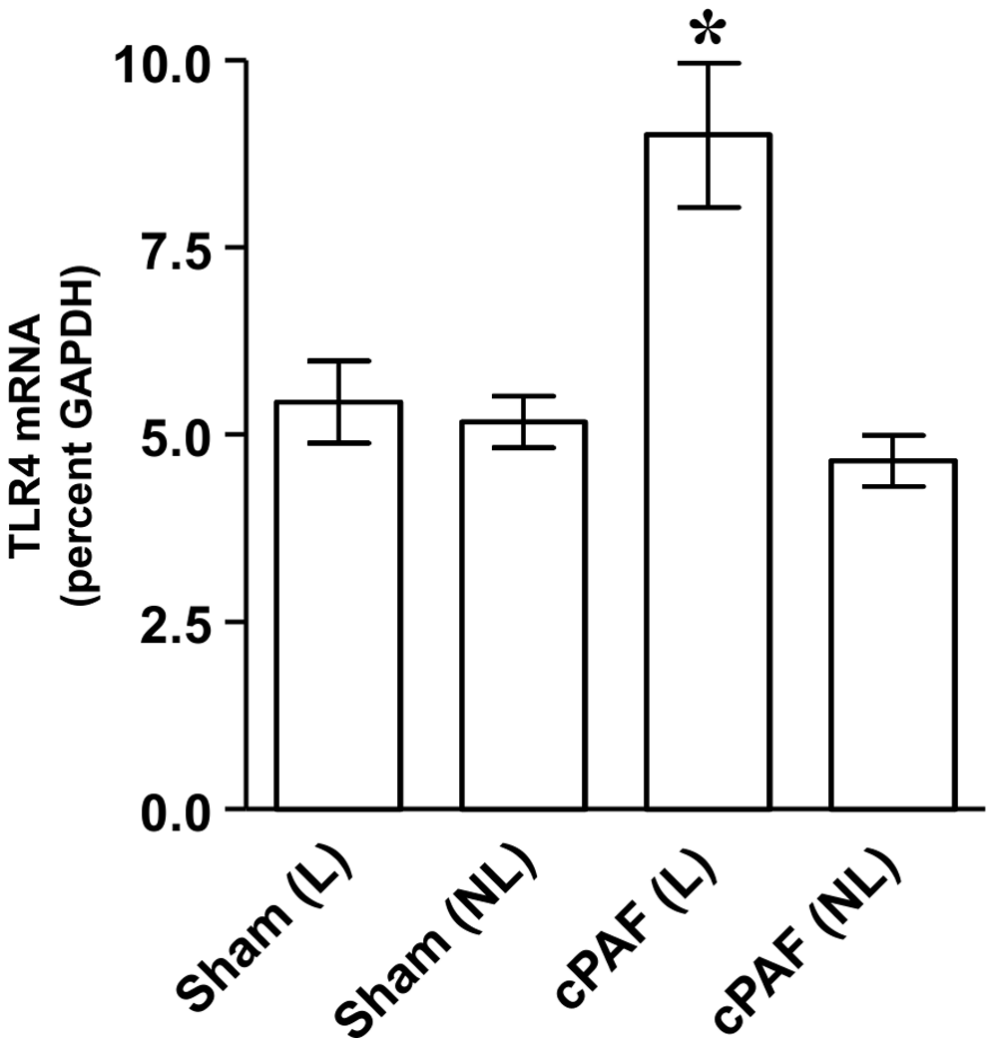
Induction of TLR4 expression by intraluminal PAF. Adult rat ileal loops were perfused with saline (sham), or with saline +10 µM carbamyl PAF for 4 hrs as indicated. Total RNA was isolated from mucosal scrapings and QRT-PCR was performed to quantify TLR4 and GAPDH mRNA copy numbers. TLR4 mRNA level is shown as % GAPDH. L indicates ileal loop mucosa, NL indicates non loop mucosa. TLR4 mRNA was significantly increased only in the mucosa that was in direct contact with PAF. * p<0.05.

TLR4 expression is very low in the mature, healthy intestinal mucosa and has been shown to be regulated by inflammatory cytokines. Our earlier studies have shown that in rodent models of necrotizing enterocolitis where PAF plays an important role, TLR4 expression is aberrantly elevated in the intestinal epithelium, implicating PAF in the regulation of TLR4 expression. In order to test whether PAF can regulate TLR4 expression, we developed a perfused ileal loop model, where the effect of intraluminal perfusion of PAF on gene expression can be directly evaluated. In this model, perfusion of PAF in the lumen of the ileum resulted in an increase of TLR4 expression in mucosal scrapings that was not observed either in the mucosa of sham-perfused animals, or in the mucosa outside of the perfused loop either in the PAF-perfused or sham perfused animals (Figure 1).

### Platelet-activating factor induces TLR4 mRNA expression and promotes IL-8 secretion in intestinal epithelial cells

**Figure 2:**
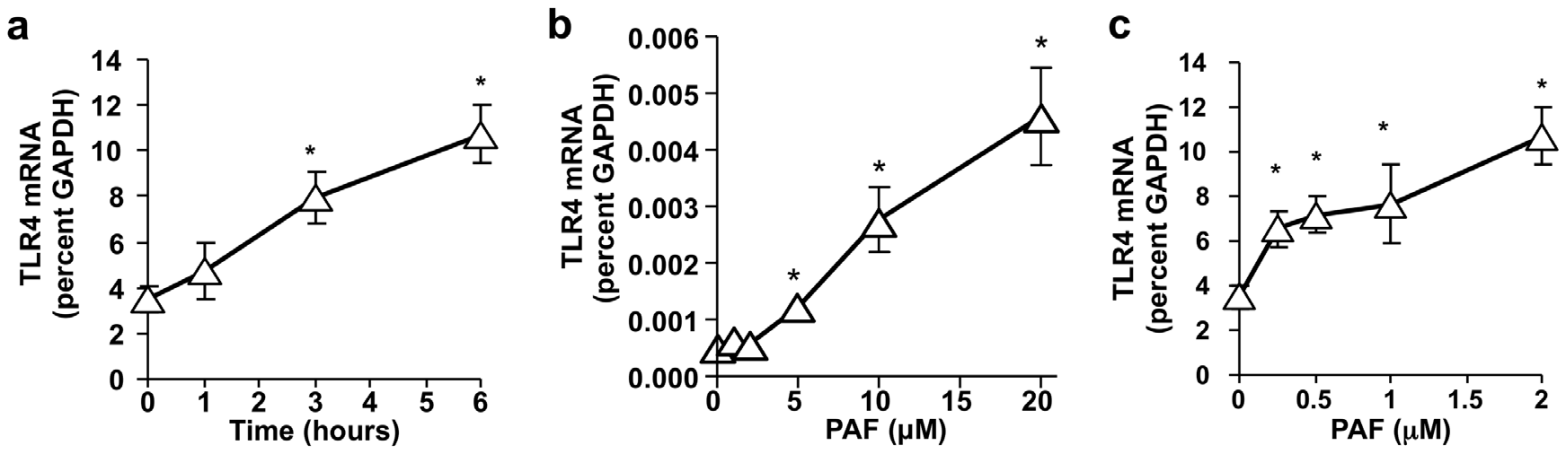
PAF-induced TLR4 expression in intestinal epithelial cells. IEC-6 (a and b) and Caco-2 (c) cells were grown in Petri dishes and treated with increasing concentrations of cPAF for for the indicated periods of time (a) or 6 h with 2 µM cPAF (b and c). cDNA copy numbers for TLR4 and GAPDH were quantified using QRT-PCR and TLR4 mRNA levels were expressed as %GAPDH. Treatment with PAF resulted in a dose- and time-dependent increase of TLR4 mRNA. * p<0.01 vs. untreated controls.

**Figure 3:**
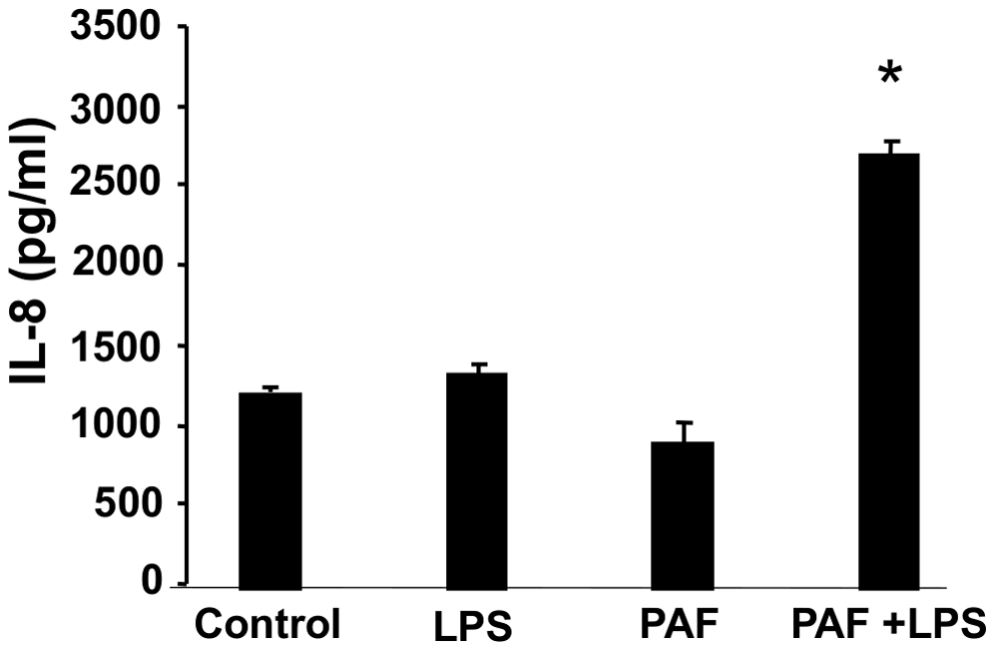
PAF increases IL-8 in intestinal epithelial cells with TLR4 ligand stimulation. Human IEC (Caco-2) were exposed to PAF 5 µM for 24 h, then stimulated for 18 h with 10 ng/ml LPS. Supernatants were harvested for IL-8 measurement by ELISA. Treatment with PAF caused a priming effect, resulting in LPS-induced IL-8 secretion by Caco-2. * p<0.05 compared to either untreated control, LPS or PAF alone.

To determine if PAF can regulate TLR4 expression specifically in epithelial cells, two experimental models were utilized. As NEC occurs more frequently in the small intestine, a murine small intestinal epithelial cell line, IEC-6, was used as our primary model. Following stimulation of cells with cPAF, we observed a dose- and time-dependent increase in TLR4 gene expression using Real Time PCR analysis (Figure 2a and b). To corroborate these results and demonstrate similar findings in human derived cells, our second experimental model utilized the Caco-2 colon cancer line. Human IEC lines recapitulate many features of normal IEC and can be used to simulate the ability of IEC to respond to pathogen associated molecular patterns (PAMPs). Several IEC lines, including the Caco-2 cell line, respond minimally to LPS because of very low TLR4 expression [20], [22], [31]. Six hours post stimulation with cPAF, we observed a subsequent dose-dependent increase in TLR4 mRNA in Caco-2 cells using Real Time PCR analysis (Figure 2c). We addressed the functionality of the induced TLR4 by exposing these cells to LPS. We found increased IL-8 secretion by Caco-2 cells exposed to 5 mM PAF for 24 h, then stimulated for 18 h with 10 ng/ml LPS as measured by ELISA. In contrast, no increase in IL-8 secretion occurred when Caco-2 cells were treated with either PAF or LPS alone (Figure 3). These results indicate that LPS could stimulate IEC secretion of IL-8, but only if TLR4 expression was first induced by treating the cells with PAF and later stimulated with TLR4 ligand.

Interestingly, the rat (IEC-6, Fig. 2b) and human (Caco-2, Fig. 2c) cell lines used possessed very different dose responses to PAF. IEC-6 cells undergo apoptosis at higher than 2 µM PAF concentrations, thus making it difficult to impossible to measure gene expression at those conditions. Additionally, the baseline TLR4 expression is significantly higher in IEC-6. Nevertheless, TLR4 expression in both cell lines is subject to regulation by PAF.

### Platelet-activating factor increases TLR4 protein expression in intestinal epithelial cells

**Figure 4:**
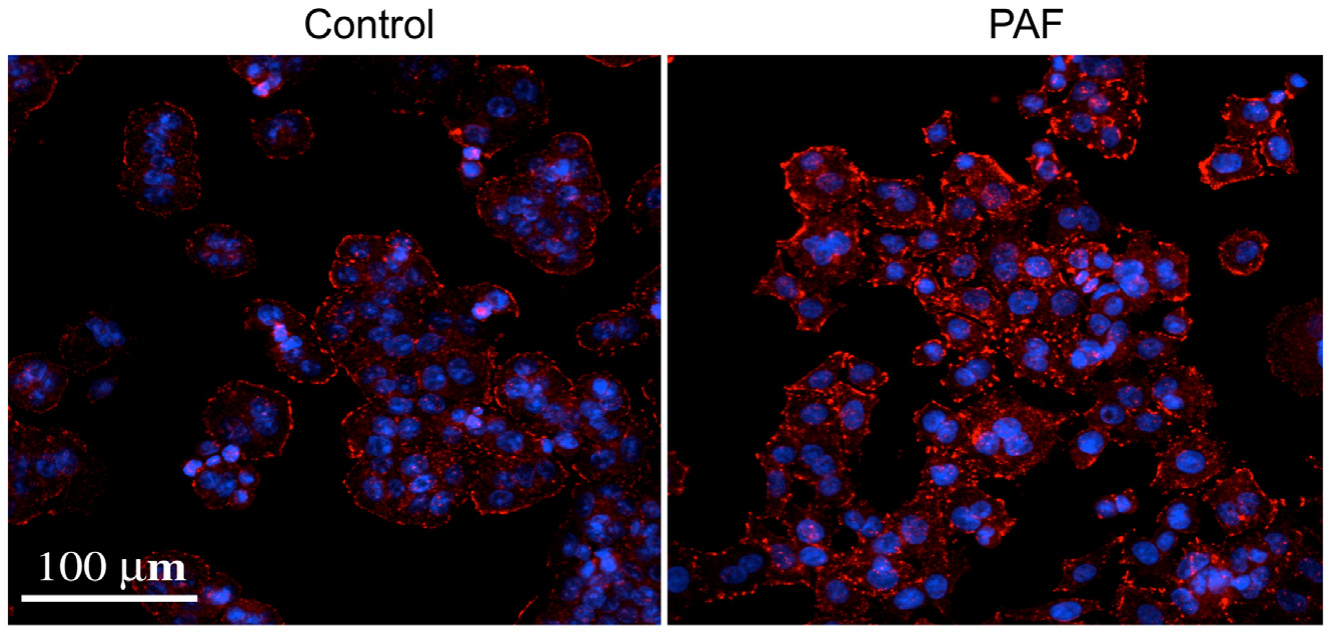
PAF induces TLR4 expression in intestinal epithelial cells. Human IEC (HT29-Cl19A cells) were grown on glass coverslips and treated without or with cPAF 5 µM for 24 h. Expression of TLR4 (shown in red) was detected using immunofluorescent microscopy (DAPI is blue). Treatment with PAF resulted in an apparently enhanced TLR4 immunoreactivity on the surface of HT29-Cl19A cells. When non-immune serum was used in place of the TLR4 antibody there was no staining detected above background (not shown).

To determine whether or not the increased mRNA of IEC following PAF stimulation leads to a corresponding increase in TLR4 protein expression, immunohistochemistry was performed. Due to extremely low expression levels in Caco-2 cells, we could not detect TLR4 and commercially available antibodies did not detect rat TLR4 in IEC-6. Upon testing various cell lines, we have found that human TLR4 is readily detected in HT29-CL19A cells. Therefore, HT29-CL19A cells were treated with or without PAF and probed with anti-TLR4 antibodies. As seen in Figure 4, there appeared to be increased TLR4 expression in cells treated with PAF as compared to unstimulated control. These data suggest that although TLR4 is weakly expressed on the surface of IEC, platelet activating factor stimulation upregulates expression of the receptor. This could potentially result in increased recognition of TLR4 ligands at the intestinal epithelial surface and subsequently lead to production of inflammatory cytokines.

### Platelet-activating factor induces TLR4 promoter activation

**Figure 5:**
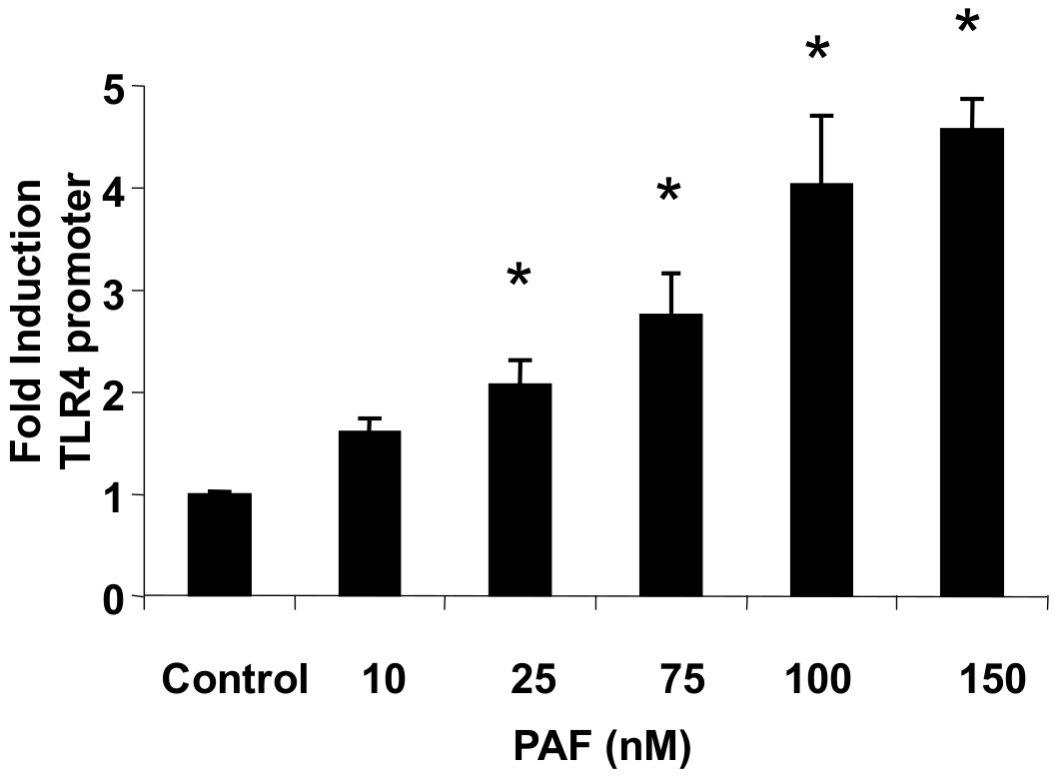
PAF induces TLR4 promoter activation. HEK293 cells transiently transfected with PAFR, human TLR4 promoter-luciferase and β-galactosidase cDNA were stimulated with cPAF (0–150 nM) for 5 h or empthy vector control. TLR4 promoter luciferase activity was measured with luciferase assay and normalized with β-gal activity. The data are expressed as fold induction of relative light units when compared with transfection of unstimulated controls. This data is representative experiment of three independent experiments performed in triplicate and *y*-error bars indicate SD. *p<0.05 compared to untreated control.

The PAF-induced changes in TLR4 mRNA and protein levels could be due to regulation of transcription or could be a result of posttranscriptional mechanisms. In order to determine whether stimulation with PAF can affect TLR4 promoter activation, we first transiently transfected HEK293 cells (which express negligible endogenous PAFR and TLR4) with a TLR4 promoter-luciferase reporter construct and PAFR cDNA. Upon stimulation with PAF, a significant dose-dependent four-fold increase in TLR4 promoter activity (compared to unstimulated empty vector control) was observed (Figure 5). Doses of PAF above 200 nM were toxic to HEK 293 cells. Control diluents at the different concentrations of PAF did not induce any luciferase activity (data not shown).

### Platelet-activating factor induces STAT3 phosphorylation and nuclear translocation in intestinal epithelial cells

**Figure 6:**
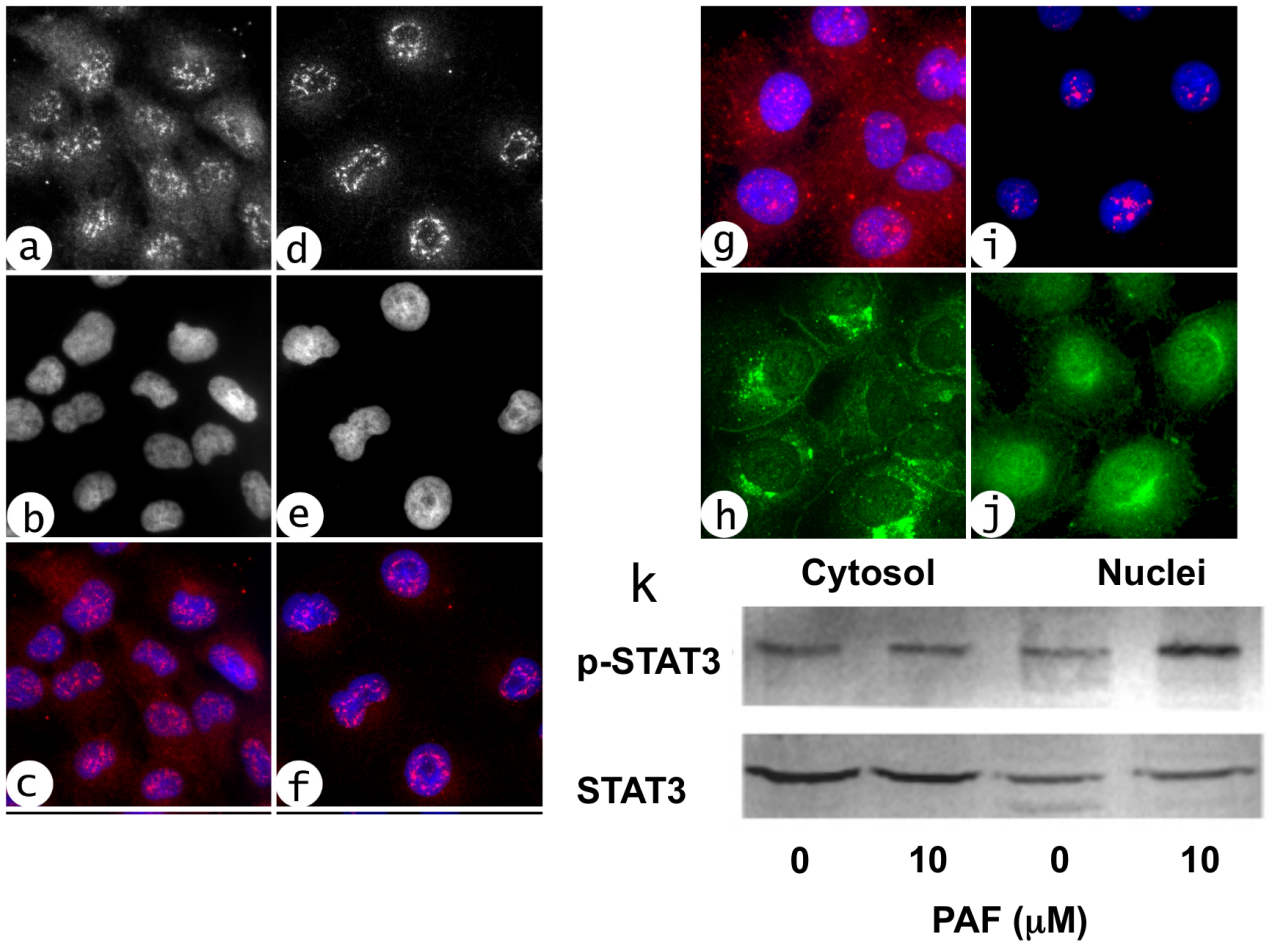
PAF-induced STAT3 translocation to nuclei in IEC-6 cells and in COS-7 cells transfected with the human PAFR. IEC-6 cells were treated without (a,b,c), or with (d,e,f) 2 µM PAF for 1 h then fixed and processed for STAT3 immunohistochemistry (a,d; c and f red) and nuclei were counterstained with Hoechst dye (b,e; c and f blue). COS-7 cells were first incubated with AD-PAFR for 24 hrs then without (g,h), or with (i,j) 1 µM PAF for 1 h. Triple label immunohistochemistry was performed with anti STAT3 (red), anti PAFR (green) and the DNA binding dye Hoechst (blue). Rat small intestinal cells (IEC-6) were treated as indicated, then Western blots were performed on cytoplasmic and nuclear fractions using antibodies against STAT3 and phorphorylated STAT3 (p-STAT3).

To begin addressing the possible signaling mechanisms that may underlie PAF-mediated regulation of TLR expression, we evaluated the potential roles of two major transcription factors regulated by PAF, STAT3 [32] and NF-κB [33], both of which have been implicated in TLR4 signaling and/or gene expression regulation [34]. We first wished to verify that PAF stimulation activated STAT3 in IEC, as was shown before in COS-7 cells. We, therefore, performed immunohistochemistry using anti-STAT3 on IEC-6 cells and COS-7 cells (which express very low levels of endogenous PAFR) transfected with human PAFR. Following PAF stimulation for 1 h, nuclear translocation of STAT3 was observed in both cell lines compared with unstimulated controls (Figure 6 a–j). To further demonstrate the effects of PAF on STAT3 in intestinal epithelial cells, STAT3 and phospho-STAT3 Western blots were performed on cytoplasmic and nuclear fractions of IEC-6 cells, following PAF stimulation. As anticipated, we observed an increase in phospho-STAT3 in the nuclear fraction following PAF stimulation but not in the unstimulated control nuclear fraction (Figure 6k). These results indicate that stimulation with PAF activates STAT3, a transcription factor that may be involved in TLR4 transcriptional regulation.

### Platelet-activating factor induced TLR4 expression is inhibited by STAT3 and NFκB inhibitors

**Figure 7:**
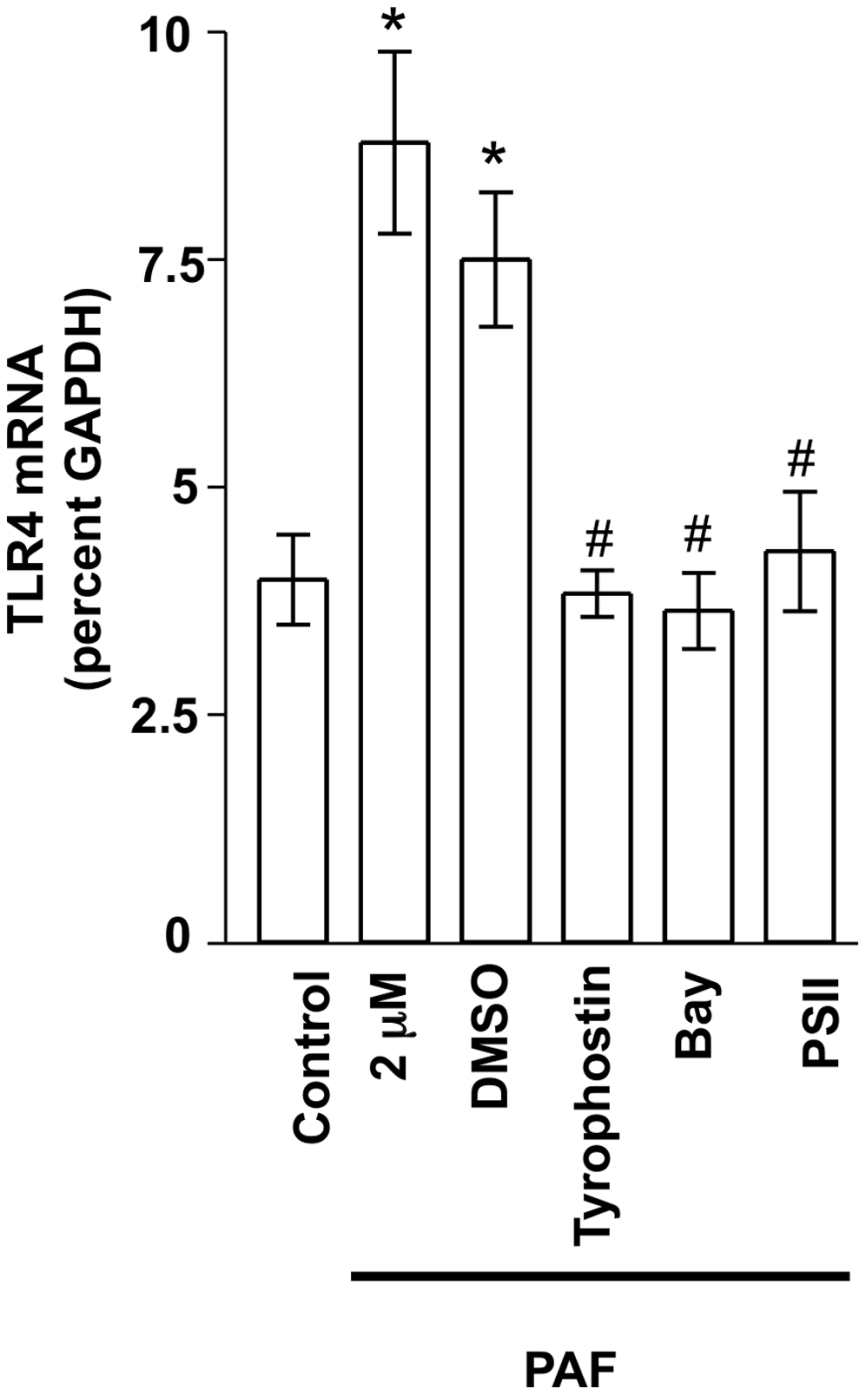
Inhibition of PAF-induced TLR4 expression by STAT3 and NFκB inhibitors. IEC-6 cells were untreated, treated with PAF (2 µM 4 h), or with PAF (2 µM 4 h) following pretreatment for 1 h with: a) DMSO (vehicle; 1∶1000; all inhibitors were dissolved in DMSO at a 1000× stock), b) Tyrophostin AG490 (25 µM), c) Bay 11–7082 (10 µM), or PSI-II (1 µM). Subsequently, total RNA was isolated and TLR4 mRNA expression was determined using GAPDH reference and quantitative real time PCR. Data shown are mean ±S.E.M. of at least n = 6 in each group. * depicts statistical significance compared to vehicle treated control of p<0.01 and # depicts statistical significance compared to PAF+DMSO of p<0.05 using ANOVA and Tukey's post hoc analysis.

To further address the potential mechanism of PAF-induced TLR4 upregulation, we pretreated IEC-6 cells with inhibitors of STAT3 and NF-κB activation, then treated the cells with PAF (2 µM 4 h), followed by QRT-PCR analysis of TLR4 expression. There was a significant reduction in TLR4 mRNA expression following PAF stimulation in those cells pretreated with Tyrphostin AG490 (an indirect STAT3 inhibitor, via JAK2 inhibition), Bay 11–7082 (an inhibitor of NFκB activation via inhibition of IκB phosphorylation), or PSI-II (an inhibitor of NFκB activation via proteasome inhibition) as compared to PAF stimulation alone (Figure 7), while DMSO, the vehicle for this compound had no effect on PAF-induced TLR4 expression. These data suggest that activation of both STAT3 and NFκB are likely involved in PAF-induced upregulation of TLR4 expression in IECs.

## Discussion

Platelet activating factor plays an important role in the initiation and/or maintenance of mucosal inflammation and intestinal injury [10]. Although PAF has been implicated in numerous diseases involving mucosal inflammation, such as asthma [35], [36], peptic ulcer[37], Crohn's disease [38], and ulcerative colitis [39], its pathogenic role is best established in neonatal necrotizing enterocolitis (NEC). We have shown that PAFR inhibition reduced the risk of NEC in a neonatal animal model using clinical risk factors for NEC (bacterial colonization, intestinal ischemia, and formula feeding) [5]. We have also shown that giving the PAF-degrading enzyme PAF-acetylhydrolase (PAF-AH) with enteral feedings prevents the initiation of experimental NEC in this rat model [7]. The intestine, particularly the distal small bowel, is at increased risk for PAF-induced inflammatory injury, since PAF-receptor gene is constitutively expressed at the highest level in the ileum compared to other organs [40]. Our previous studies have shown that the development of NEC in a rodent model is preceded and underlined by exaggerated enterocyte apoptosis [41] and that PAF is a potent inducer of enterocyte apoptosis in tissue culture [42]. However, while the role of PAF has been established in this NEC neonatal rat model, the mechanisms by which PAF contributes to NEC are not completely understood.

TLRs are important in the protection of the host. They initiate the innate immune response modulate the adaptive immune response to bacteria and viruses. TLRs recognize PAMPs that may derive either from bacteria, viruses or fungi and initiate signaling that results in expression and release of inflammatory cytokines. We and others [20], [22], [34], [43], [44] have shown that IEC of the mature, healthy small and large intestine express low levels of TLR4 and MD-2 and respond poorly to LPS, but TLR4 and MD-2 are abnormally upregulated in inflammatory bowel disease [34]. The very low level of expression of and subdued signaling via TLRs may represent an adaptation of IEC to prevent collateral tissue damage from an unnecessary inflammatory response to commensal organisms. Ongoing signaling between commensals and TLRs has an important beneficial effect on the maintenance of normal barriers in the intestine and enhances protection and repair from injury [45]. We have also demonstrated in an animal model of acute colitis using dextran sulfate sodium that TLR4, through the adapter protein MyD88, is important in the intestinal response to epithelial injury and limiting bacterial translocation [46]. We know that TLRs are expressed on fetal enterocytes [47]. More directly, we have recently shown in an established neonatal rat model and a novel neonatal murine model of NEC, that bacteria and TLR4 play a significant role in experimental NEC, likely via an interaction of intraluminal bacteria and aberrantly overexpressed TLR4 in enterocytes [16].

Our current study sought to provide a link between PAF and TLR4 expression in explaining how their interaction could shed light on the pathogenesis of NEC. We found that PAF is able to upregulate the normally low TLR4 expression in rat intestinal epithelium *in vivo* and in both human and rat IEC in tissue culture. Furthermore, priming with PAF directly led to enhanced LPS-induced IL-8 secretion, thus showing the functional effect of increased TLR4 expression. Whether the priming effect of PAF on LPS-induced IL-8 secretion can be explained solely by the PAF-induced higher level TLR4 expression or whether PAF also had an effect on TLR4 signaling is yet to be determined.

We show that perfusion of PAF in the lumen of the ileum in this experimental model results in an increase of TLR4 expression in mucosal scrapings that is not seen either in the mucosa of sham-perfused animals or in the mucosa outside of the perfused loop either in the PAF-perfused or sham perfused animals. These observations imply that luminal exposure of PAF can lead to changes in TLR4 expression and signaling. Indeed, several studies have shown that in this rodent model of NEC PAF levels as well as PAF-synthesizing enzyme PLA2 are increased in tissue homogenates and are secreted into the lumen [10]. Furthermore, in the human preterm infant with NEC, luminal stool levels of PAF are increased compared to those without NEC [48].

Our findings provide initial mechanistic explanations for PAF-induced TLR4 expression. TLR4 expression may be primarily due to PAF, or to a downstream molecule released in response to PAF. In trying to further define the potential cascade initiated by PAF-induced TLR4 expression, we focused on the roles of two transcription factors, STAT3 and NF-κB. Consistent with previous reports in other cell lines that PAF stimulation results in phosphorylation and nuclear translocation of STATs [32], [49], [50], we demonstrated that STAT3 is phosphorylated upon PAF stimulation in intestinal epithelial cells, and that this leads to nuclear translocation. Although the TLR4 promoter region has no direct STAT3 binding site, its PU.1 binding site is considered to be one of its main regulatory elements and STAT3 has been shown to be capable of activating PU.1 [51]. It is also likely that TLR4 upregulation reflect a secondary effect of a downstream STAT3 product. NF-κB is both a downstream effector of TLR signaling and it is an upstream regulator of TLR4 expression, making this transcription factor a central molecule in inflammatory regulation. PAF has been shown to activate NF-κB in enterocytes *in vivo* [52] and in several other cell types in tissue culture [33], [53]. In light of PAF's ability to activate NF-κB and given that the TLR4 promoter contains NF-κB binding sites [29], it is not surprising that we observed a dependence of PAF-induced TLR4 gene expression on NF-κB activation. Future experiments will elucidate the exact mechanisms of STAT3 involvement, and possibly other factors, in PAF mediated transcriptional activation of TLR4 and will investigate experimental models where PAF is neutralized.

In summary, PAF has been recognized to play an important role in the development of the intestinal inflammatory injury seen in NEC. Bacteria have also been implicated in the pathogenesis of neonatal NEC, and the activation of abnormally expressed TLRs at the intestinal epithelium is central to this process. Results here suggest that one possible link between these is that PAF stimulation triggers dysfunctional innate immune signaling that over-reacts to bacterial ligands from gut commensals, and thus leads to inflammation and associated collateral tissue damage. Our studies thus contribute to our understanding of how the intestinal epithelium participates in the innate immune response to PAF and bacterial ligands in neonatal NEC, and opens the door for future experimental studies and possibly even targeted therapies to limit disease, or perhaps even to prevent NEC in at risk patients.
